# *LIN28A* Expression Reduces Sickling of Cultured Human Erythrocytes

**DOI:** 10.1371/journal.pone.0106924

**Published:** 2014-09-04

**Authors:** Jaira F. de Vasconcellos, Ross M. Fasano, Y. Terry Lee, Megha Kaushal, Colleen Byrnes, Emily R. Meier, Molly Anderson, Antoinette Rabel, Raul Braylan, David F. Stroncek, Jeffery L. Miller

**Affiliations:** 1 Molecular Genomics and Therapeutics Section, Molecular Medicine Branch, National Institute of Diabetes and Digestive and Kidney Diseases, National Institutes of Health, Bethesda, Maryland, United States of America; 2 Center for Cancer and Blood Disorders, Children's National Medical Center, Washington, District of Columbia, United States of America; 3 Department of Laboratory Medicine, Clinical Center, National Institutes of Health, Bethesda, Maryland, United States of America; 4 Cell Processing Section, Department of Transfusion Medicine, Clinical Center, National Institutes of Health, Bethesda, Maryland, United States of America; Southern Illinois University School of Medicine, United States of America

## Abstract

Induction of fetal hemoglobin (HbF) has therapeutic importance for patients with sickle cell disease (SCD) and the beta-thalassemias. It was recently reported that increased expression of LIN28 proteins or decreased expression of its target *let-7* miRNAs enhances HbF levels in cultured primary human erythroblasts from adult healthy donors. Here *LIN28A* effects were studied further using erythrocytes cultured from peripheral blood progenitor cells of pediatric subjects with SCD. Transgenic expression of *LIN28A* was accomplished by lentiviral transduction in CD34(+) sickle cells cultivated *ex vivo* in serum-free medium. *LIN28A* over-expression (*LIN28A*-OE) increased HbF, reduced *beta (sickle)-globin*, and strongly suppressed all members of the *let-7* family of miRNAs. *LIN28A*-OE did not affect erythroblast differentiation or prevent enucleation, but it significantly reduced or ameliorated the sickling morphologies of the enucleated erythrocytes.

## Introduction

Sickle Cell Disease (SCD) and the beta-thalassemias are among the most prevalent genetic disorders worldwide with high levels of morbidity and mortality [1], [2]. Our understanding of these beta-hemoglobinopathies at both the clinical and molecular levels has increased greatly in recent decades [3]. However, despite a homogeneous genetic mutation, clinical outcomes and response to therapy vary widely among patients with SCD [4].

Pharmaceutical induction of fetal hemoglobin (HbF) is an effective approach toward therapy [1]. HbF levels of 20% or greater are associated with improvements in the SCD patients' clinical status [5]. Therefore, manipulation of HbF levels in human erythrocytes remains an intense topic of benign hematology research. As a result, significant knowledge regarding the molecular mechanisms underlying HbF regulation has been uncovered. For instance, gene linkage studies associated *BCL11A*, *HSB1L*-*MYB* and *HBB* regions in the genome with increased HbF expression in adults [6]–[9], and suppression of *BCL11A* causes an increase in HbF levels and reverses the SCD phenotype in model systems [10], [11].

Recently, LIN28A and LIN28B proteins as well as *let-7* microRNAs (miRNAs) were shown to cause a partial reversal of ontogeny-related changes in the human erythroid phenotype including increased expression of HbF in adult cells [12]. The highly conserved RNA-binding LIN28 proteins are known to control developmentally-timed events in multicellular organisms by negatively regulating the biogenesis of *let-7* miRNAs [13]. Human *let-7* miRNAs demonstrate increased expression in adult human reticulocytes when compared to cord blood reticulocytes [14].

Here we study erythrocytes cultured from purified CD34(+) cells from children with SCD to further explore the potential for transgenic erythroid *LIN28A* expression to decrease *let-7* miRNAs levels and increase HbF expression. We also report a novel assay for exploring the effects of increased HbF expression upon the sickling phenomenon using purified erythrocytes generated in serum-free culture.

## Methods

### Ethics Statement

Institutional Review Board approval for the study and the informed consent process was obtained from Children's National Medical Center and the National Institute of Diabetes and Digestive and Kidney Diseases. Written informed consent was obtained from the parent or legal guardian for all research participants less than 18 years of age and from all research subjects 18 years of age or older prior to participation in this study. Written assent was obtained from all participants between the ages of 7 and 17 years of age.

### Patients and samples

Discarded whole blood from partial manual exchange transfusions was collected from five pediatric research subjects with HbSS genotype (ages 9–16 years) who were at steady state and not being treated with hydroxyurea. The partial manual exchange transfusions were performed as primary (Donors 1–4) or secondary (Donor 5) stroke prophylaxis every 4 to 5 weeks. The chronic transfusion regimens had been in place for a range of 3 to 11 years at the time of this study. Patient's clinical features are described in Table 1. After the RBCs were lysed (ACK Lysing Buffer, Lonza, Walkersville, MD), CD34(+) cells were isolated using CD34 antibodies conjugated to magnetic microbeads (CliniMACS CD34 Reagent, Miltenyi Biotec GmbH, Bergisch Gladbach, Germany) and a magnetic column (CliniMACS Instrument, Miltenyi Biotec).

**Table 1:** Clinical features from the five HbSS patients used in this study.

Donor #	Gender	Age (years)	Hgb (g/dL)	%S	%F	%A	%A2
1	M	15	9.9	37.2	4.1	55.8	2.9
2	M	13	9.8	36.3	3.4	57.1	3.2
3	M	9	8.5	20.0	1.3	76.1	2.6
4	M	9	8.7	34.3	3.1	59.6	3.0
5	M	16	10.5	38.3	7.1	51.3	3.3

### Cell culture

E*x vivo* culture was performed in a 3-week serum-free system consisting of three phases (phase I from day 0 to 7, phase II from day 7 to 14 and phase III from day 14 to 21) as previously described [12]. Throughout phase III, cells were kept in a 2% oxygen environment.

### Recombinant viral transduction

Human *LIN28A* over-expression and empty vector control lentiviral particles were purchased from Qiagen (Valencia, CA). On culture day 3 of phase I, CD34(+) cells were transduced with *LIN28A* viral particles (estimated MOI 12). After 24 hours, puromycin (Sigma Aldrich, St. Louis, MO) was added to the culture. On culture day 7, cells were transferred to phase II medium containing EPO and puromycin until culture day 9. After culture day 9, cells were cultivated at the conditions previously described without puromycin.

### Flow cytometry analyses

On days 14 and 21, erythroid differentiation was assessed with antibodies directed against CD71 and glycophorin A (Invitrogen, Carlsbad, CA) using the BD FACSAria I flow cytometer (BD Biosciences, San Jose, CA) as previously described [15]. Enucleation was quantitated by thiazole orange (TO) staining (Sigma) on day 21. Enucleated sickle erythrocytes [TO(−) population] were sorted and imaged as described below. Flow cytometric analyses of HbF were performed in cells fixed with paraformaldehyde and stained with antibody against HbF as previously reported [16].

### Quantitative PCR and Western analyses

Q-RT-PCR assays and Western analyses were performed as previously described [12], [14], [17].

### HPLC for sickle and fetal hemoglobins

Samples for HPLC analysis were prepared and analyzed as previously described [15].

### Low oxygen exposure and assessment of cellular morphologies

Cells were cultured in a 3-week serum-free system as previously described [12]. During the final week in culture, cells were incubated in a 2% oxygen environment consistent with the lower oxygen range estimated for human bone marrow [18]. Initial experiments using cells from healthy volunteers demonstrated that 2% oxygen conditions significantly increased enucleation compared to matched cells cultured in a 21% environment (21% oxygen: 29.5±10.5%; 2% oxygen: 44.0±13.9%; p = 0.019; Figure S1). Enucleated cells detected by thiazole orange staining were sorted directly into wells containing culture medium [duplicate plates for control and *LIN28A* over-expression (*LIN28A*-OE) transductions]. Sorted cells as well as control erythrocytes from individuals with HbSS were incubated for 16 hours at 2% oxygen prior to imaging studies. All images were taken within three minutes after removal from 2% oxygen without further manipulation of the cells. Cell imaging was accomplished using an inverted microscope (32X magnification) equipped with a Zeiss AxioCam MRc5 camera (Carl Zeiss, Oberkochen, Germany). Four random fields were imaged from each well, and cellular morphologies were scored by blinded observers.

### Statistical analysis

Mean ± SD values were used for calculating statistical significance by Student's t-test.

## Results

### *LIN28A* suppresses the *let-7* family of miRNAs in human CD34(+) sickle cells

**Figure 1:**
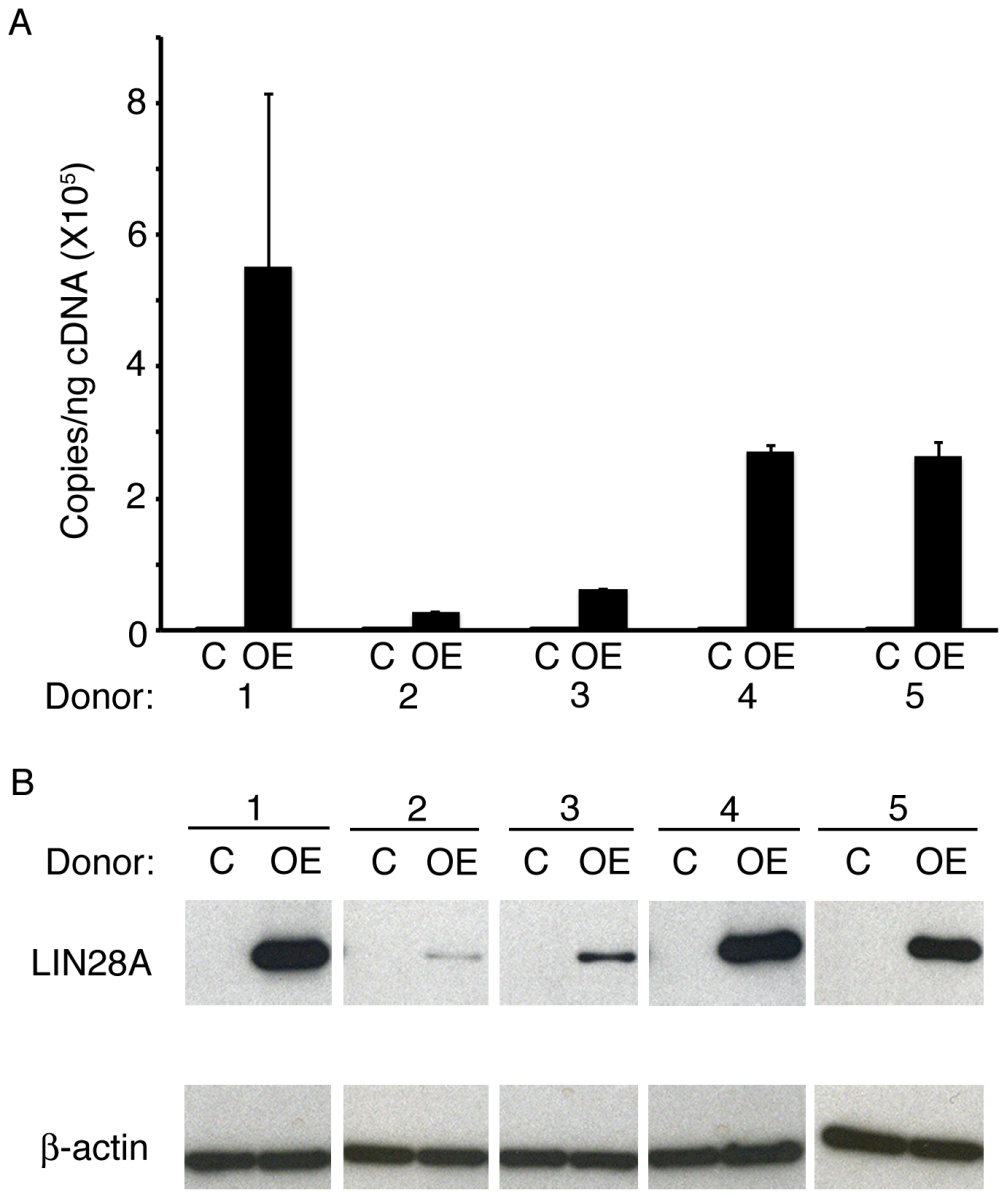
*LIN28A* over-expression in human sickle erythroblasts was confirmed by (A) Q-RT-PCR quantitation of copy number *per* nanogram of complementary DNA (cDNA) (copies/ng cDNA) and (B) Western blot analysis. Analyses were performed at culture day 14. Open bars represent control and black bars represent *LIN28A* over-expression. C: empty vector control; OE: *LIN28A* over-expression.

**Figure 2:**
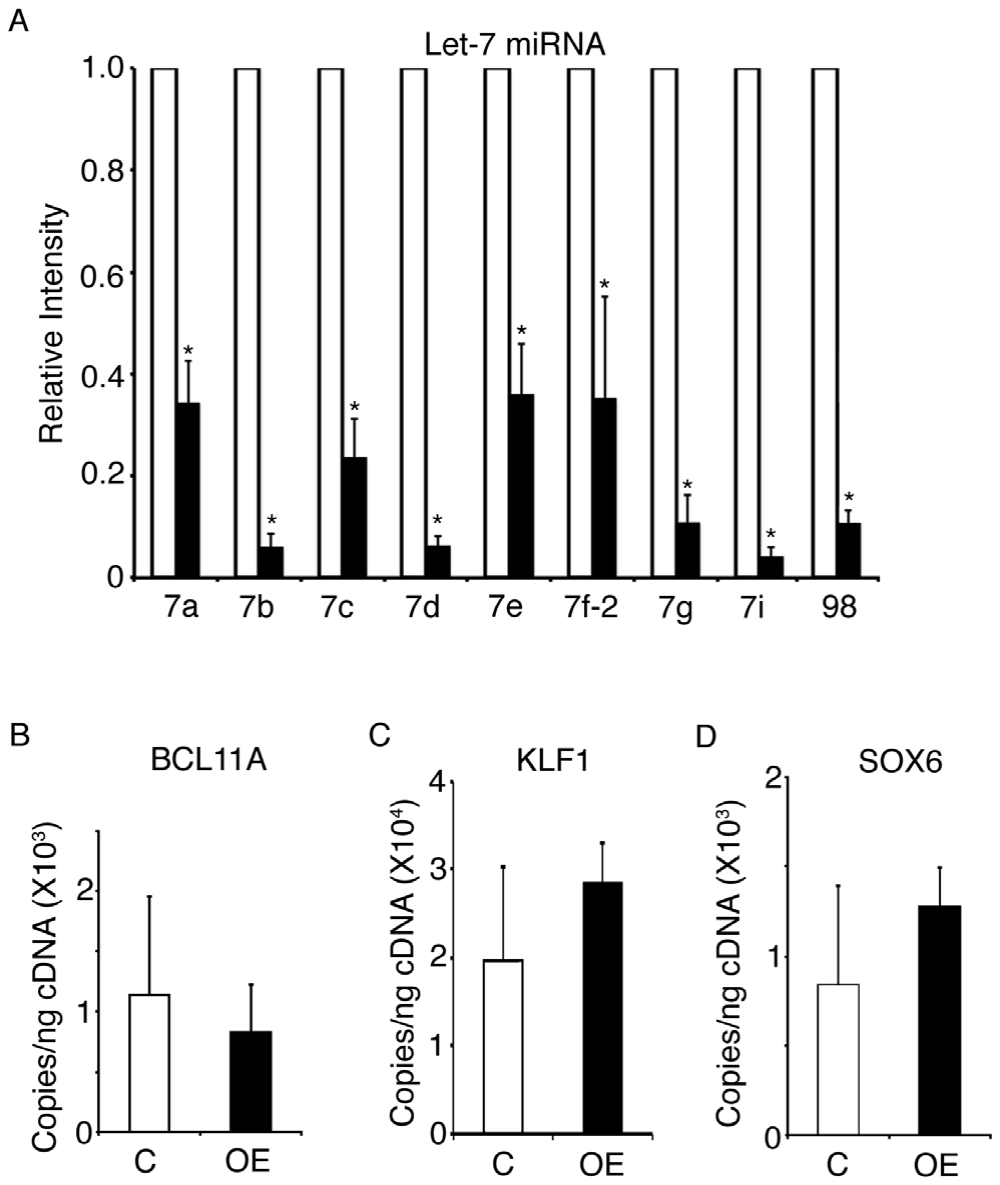
*LIN28A* over-expression strongly suppresses all members of the *let-7* family of miRNAs in cultured human sickle erythroblasts. **(A)** *LIN28A* over-expression compared to control samples in the relative expression levels of the *let-7* family of miRNAs. The miRNA relative expression levels (y-axis) in the control cells were defined as a level of one for comparison. *LIN28A* over-expression compared to control samples in the mRNA expression levels of **(B)** *BCL11A*, **(C)** *KLF1* and **(D)** *SOX6*. Analyses were performed at culture day 14. Open bars represent control and black bars represent *LIN28A* over-expression. Mean value ± SD of five independent research subjects for each condition. P values were calculated using Student's t-test. C: empty vector control; OE: *LIN28A* over-expression. *p<0.05.

Increased LIN28 in cultured erythroblasts from adult healthy donors causes increased gamma-globin gene and protein expression as they differentiate in culture [12]. As a result, the HbF content of the enucleated erythrocytes that are produced is also elevated. To explore these effects in primary sickle cells, we investigated *LIN28A*-OE using erythroblasts from five children with SCD. CD34(+) cells were transduced with a *LIN28A* encoding lentivirus, and *LIN28A*-OE was confirmed by Q-RT-PCR (control: 8.6E+00±8.1E+00 copies/ng, *LIN28A*-OE: 2.3E+05±2.1E+05 copies/ng, p = 0.033) and Western blot analyses (Figure 1). *LIN28A*-OE did not affect the expression of its homolog *LIN28B* as assessed by Q-RT-PCR on culture day 14 (data not shown). As expected, *LIN28A*-OE strongly suppressed the levels of all *let-7* miRNA family members, with average reductions from 64–96% for *let-7a*, *let-7b*, *let-7c*, *let-7d*, *let-7e*, *let-7f-2*, *let-7g*, *let-7i* and *miR-98* (Figure 2A). *LIN28A*-OE mediated changes in the expression of *BCL11A*, *KLF1* and *SOX6*, erythroid transcription factors involved in the regulation of HbF [10], [19], were not statistically significant (Figure 2B–D). Examination of *BCL11A* expression in each donor's cells revealed variable changes associated with *LIN28A*-OE when compared with controls (Figure S2).

### *LIN28A* regulates fetal hemoglobin expression but does not affect erythroid differentiation or enucleation of cultured sickle erythrocytes

**Figure 3:**
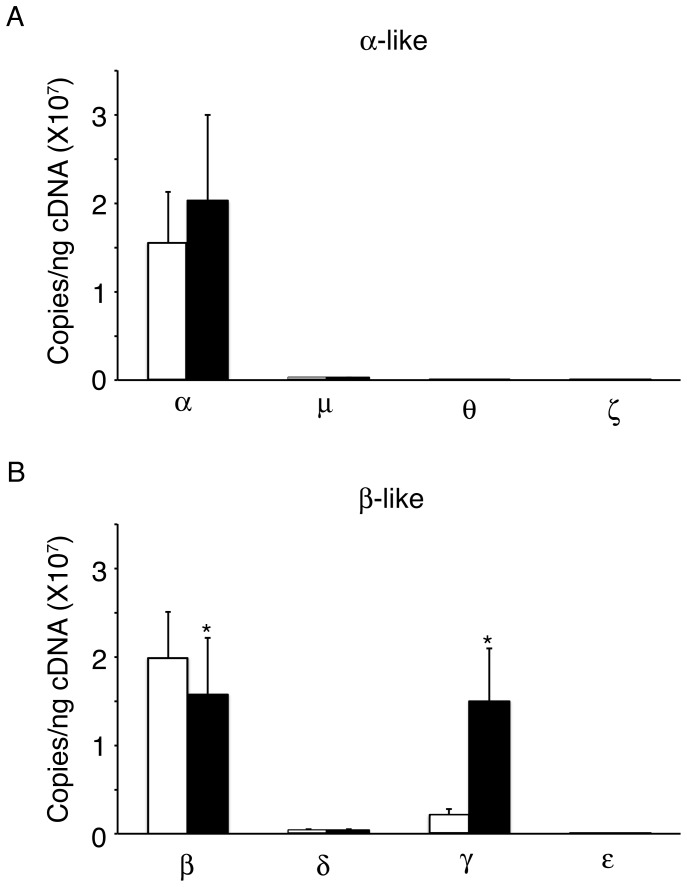
*LIN28A* regulates *gamma-globin* mRNA levels in cultured human sickle erythroblasts. *LIN28A* over-expression compared to control samples in the mRNA expression levels of **(A)** *alpha*-, *mu*-, *theta*- and *zeta*-*globins* and **(B)** *beta*-, *delta*-, *gamma*- and *epsilon*-*globins*. Analyses were performed at culture day 14. Open bars represent control and black bars represent *LIN28A* over-expression. Mean value ± SD of five independent research subjects for each condition. P values were calculated using Student's t-test. *p<0.05.

Expression of the globin genes demonstrated no major differences in *alpha*-, *mu*-, *theta*-, *zeta*-, *delta*- and *epsilon*-*globins* mRNA levels among *LIN28A*-OE cells (Figure 3A). However, g*amma*-*globin* mRNA expression levels increased significantly in *LIN28A*-OE samples (control: 2.0E+06±7.0E+05 copies/ng, *LIN28A*-OE: 1.5E+07±6.0E+06 copies/ng, p = 0.006), and *beta (sickle)*-*globin* mRNA decreased in *LIN28A*-OE samples (control: 2.0E+07±5.2E+06 copies/ng, *LIN28A*-OE: 1.6E+07±6.3E+06 copies/ng, p = 0.024; Figure 3B).

**Figure 4:**
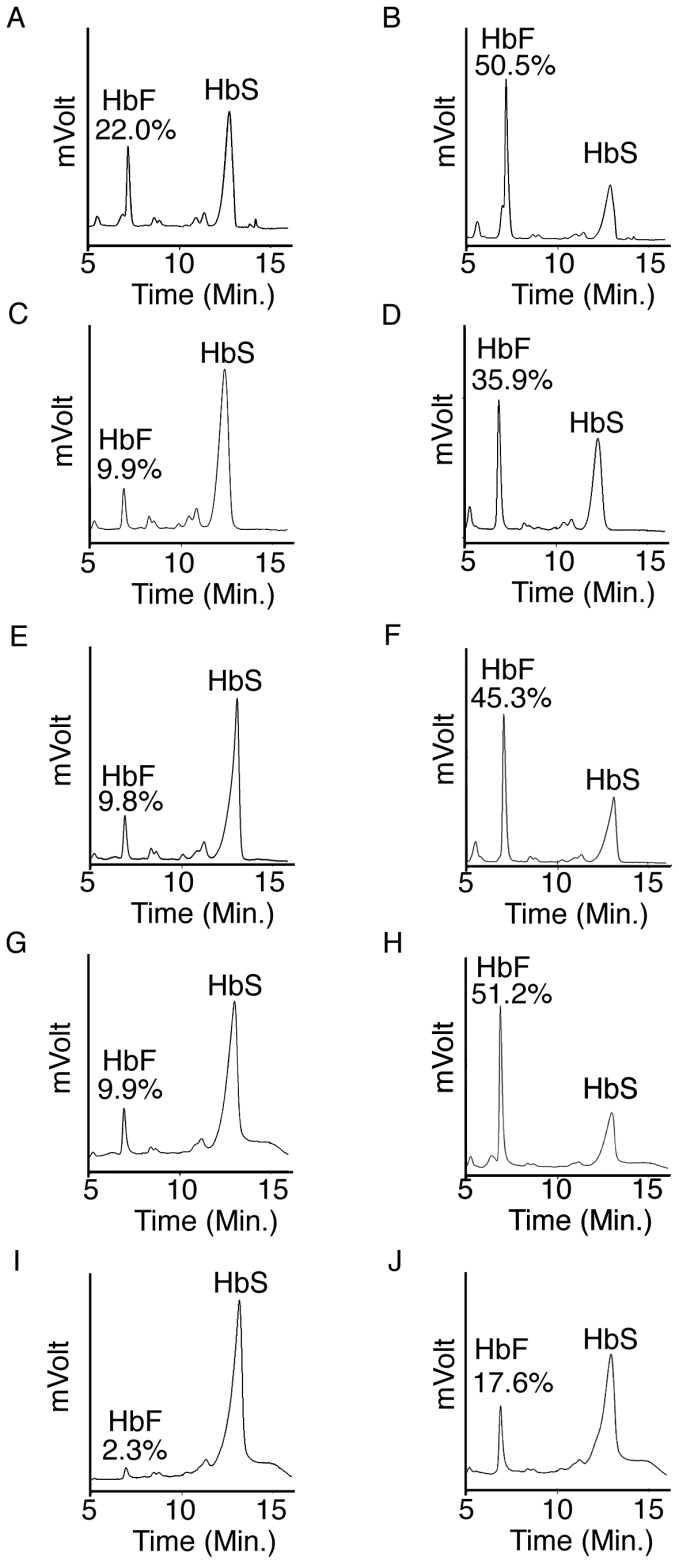
*LIN28A* regulates fetal hemoglobin levels in cultured human sickle erythroblasts. HPLC analysis of hemoglobin from control and *LIN28A*-OE samples were performed at culture day 21. Donor 1 **(A)** Control and **(B)** *LIN28A*-OE, Donor 2 **(C)** Control and **(D)** *LIN28A*-OE, Donor 3 **(E)** Control and **(F)** *LIN28A*-OE, Donor 4 **(G)** Control and **(H)** *LIN28A*-OE and Donor 5 **(I)** Control and **(J)** *LIN28A*-OE. HbF and HbS peaks are labeled with the ratio of HbF/HbF+HbS expressed as a percentage above each HbF peak (y-axis, mVolts; x-axis, elution time in minutes).

Consistent with the changes in mRNA levels, *LIN28A*-OE increased the percentage of HbF compared to controls. Increased HbF was detected by HPLC for each donor (Figure 4A–J). The mean increase in HbF was statistically significant (control: 10.8±7.1%, range 2.3 to 22%; *LIN28A*-OE: 40.1±14.0%, range 17.6 to 51.2%; p = 0.003). The absence of HbA or other major peaks confirmed the HbSS phenotype. Fetal hemoglobin staining demonstrated a pancellular shift in HbF staining when compared to the isotypic controls (Figure S3). A bimodal distribution of HbF-mediated fluorescence intensity was noted in the controls. By comparison, *LIN28A*-OE caused a significant increase of cells demonstrating higher level fluorescence (control: 45.0±17.6%, *LIN28A*-OE: 66.5±18.6%, p = 0.007).

According to flow cytometry analyses of transferrin receptor (CD71) and glycophorin A (GPA), erythroblast differentiation was not affected by *LIN28A*-OE (Figure S4A). Furthermore, thiazole orange (TO) staining, showed equivalent enucleation achieved between the *LIN28A*-OE cells and the control transductions at culture day 21 (*LIN28A*-OE enucleation 40.8±17.0% compared to control 49.9±23.4%, p = 0.19; Figure S4B). Upon completion of the culture period (day 21), cell counts in *LIN28A*-OE and control transductions were equivalent (data not shown). Taken together, these data show that transgenic expression of *LIN28A* in erythroblasts from pediatric subjects with sickle cell anemia regulates both fetal and *beta (sickle)-globin* expression with comparable *ex vivo* differentiation of the cells.

### LIN28A reduces hypoxia-related sickling

**Figure 5:**
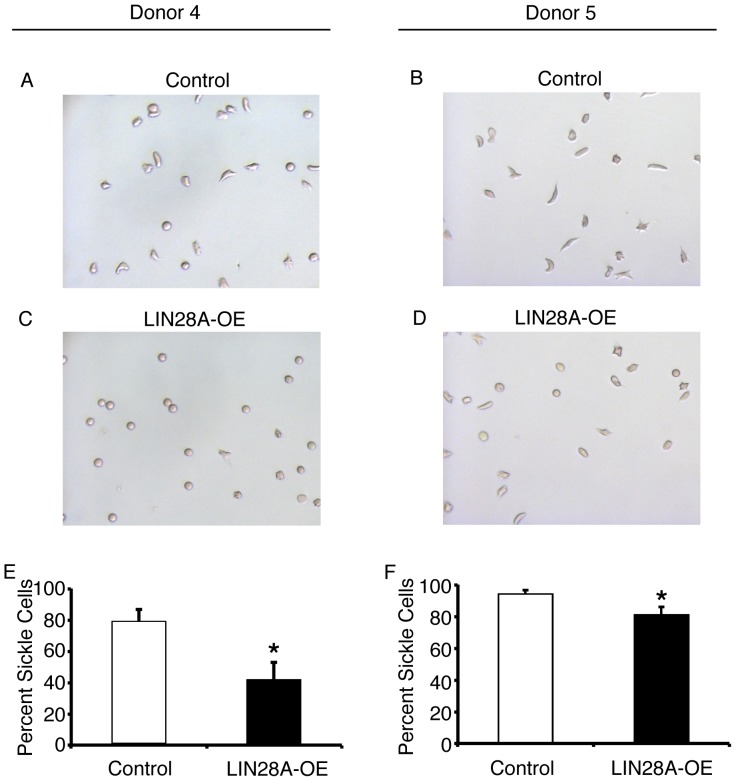
*LIN28A* over-expression ameliorates abnormal morphologies associated with sickle mature erythrocytes. Cell images are representative from two independent research subjects as follows: Donor 4 **(A)** control and **(C)** *LIN28A*-OE, and Donor 5 **(B)** control and **(D)** *LIN28A*-OE. Cells were scored according to round *versus* variable sickle morphologies (non-round). Average cell counts of variable sickle morphologies from Donor 4 **(E)** and Donor 5 **(F)**. Error bars denote ± SD of 8 fields for each condition *per* donor. P values were calculated using Student's t-test. Control: empty vector control; *LIN28A*-OE: *LIN28A* over-expression. *p<0.05.

Based upon the demonstration of robust enucleation, we next determined whether sickle cell morphologies could be identified among the cultured erythroblasts. The assay was developed using cells from the first three donors, and the morphologies were scored for comparison using Donor 4 and Donor 5 cells. Microscopic examination showed morphologies that were characteristically similar to those reported from human blood including elongated, maple-leaf, and the classic “sickle” shaped cells [20] (See Figure 5 and Figure S5). By comparison, images from healthy HbAA donor cells produced round morphologies in culture (Figure S6). As a result of these differences, the cellular populations were scored according to two general categories: round cells (no evidence of sickle morphology) *versus* cells with non-round shapes defined in this study as possessing variable sickle morphologies. Further attempts to subcategorize were not made due to the broad range of abnormal morphologies. According to this scoring method, control erythrocytes obtained from HbSS donors' peripheral blood that were maintained under the same 2% oxygen environment in culture medium demonstrated sickled morphologies in 88.8±5.4% of the cells.

The same morphology scoring method (round *versus* non-round) was utilized to quantitate *LIN28A*-OE effects upon the morphological sickling phenomenon. Images from the control and *LIN28A*-OE transductions were assigned random numbers, and then scored by blinded observers. Cellular debris or non-hemoglobinized cellular ghosts (less than 10% of particles) were not scored. The percentages of cells from each donor that demonstrated variable sickled morphologies (non-round) were compared between *LIN28A*-OE and control transductions. Representative fields are shown in Figure 5A–D (all microscopic fields are shown in Figure S5). *LIN28A*-OE caused significant reductions in the variable sickle morphologies in both donors' cells. In Donor 4, *LIN28A*-OE caused HbF expression to increase in the cells to 51.2% (*versus* control HbF 9.9%), with a marked reduction in the variable sickle morphologies of the cultured erythrocytes (control: 79±7.8%, *LIN28A*-OE: 41.6±11.1%, p = 0.00004; Figure 5E). In Donor 5 cells, *LIN28A*-OE caused increased HbF levels to 17.6% (*versus* control HbF 2.3%), and caused a less intense reduction in the variable sickle morphologies (control: 94.1±2.3%, *LIN28A*-OE: 81.8±4.4%, p = 0.0003; Figure 5F). While both donors demonstrated decreases in sickling, the more robust reduction in Donor 4 cells occurred in association with the greater increase in HbF.

## Discussion

The *lin28* gene was first described in the nematode *Caenorhabditis elegans* as a heterochronic gene that regulates timing and sequence of developmental events [21]. Two homologs of the *lin28* gene – *LIN28A* and *LIN28B* – have been identified in humans and were correlated with the pluripotency of stem cells [22], differentiation of skeletal muscle cells [23], and developmental timing characteristics such as variation in height [24], timing of puberty [25], [26] and age at natural menopause [26]. *LIN28* genes are known to regulate the *let-7* family of miRNAs, and the expression of *LIN28* transcripts is associated with the inhibition of *let-7* [13]. The *LIN28*-*let-7* axis is highly conserved across evolution, serving as a regulator of cell growth and development in a cellular/tissue specific manner. In erythroid biology, the *let-7* family of miRNAs was associated with the developmental transition from fetal to adult erythropoiesis, since several members of the *let-7* family are up-regulated in adult compared to cord blood human reticulocytes samples [14]. Recently, expression of either LIN28 protein in adult erythroblasts caused reductions in *let-7* miRNAs as well as increases in HbF expression [12]. Here our studies with *LIN28A* over-expression demonstrate similar effects in cultured human sickle cells. *LIN28A*-OE was also shown to reduce the cell-sickling phenomenon in these cultured cells.

Potential effects of *LIN28A*-OE upon the expression of *BCL11A*, *SOX6* and *KLF1* were also studied [12], [27]. *BCL11A* was previously associated with the human genetic variation in HbF levels [6], [7], [9], [24], the regulation of HbF in adult erythroid cells [10], and the amelioration of the SCD phenotype in adult mice models of the disease [11]. In SCD patients, *BCL11A* has been associated with the baseline levels of fetal hemoglobin [28] and was down-regulated by hydroxyurea treatment in early reticulocytes [29]. In cooperation with *BCL11A*, the transcription factor *SOX6* was also reported to occupy the human beta-globin cluster and play a role in regulating HbF in adult human erythroid cells [30]. *KLF1* was identified as a regulator of *BCL11A* expression in human erythroid cells [19]. Significant reductions in *BCL11A* expression upon the transgenic over-expression of LIN28 were previously detected in cells from healthy donors [12]. In this study, the suppression of *BCL11A* expression was not consistent among the 5 donors (Figure S2), and *LIN28A* over-expression with *let-7* miRNAs suppression did not significantly change the mean expression levels of *BCL11A, KLF1* or *SOX6* (Figure 2B–D). Further, all the samples demonstrated increased levels of HbF, but those increases were not well correlated with the levels of *LIN28A* over-expression (compare Figures 1 and 4). These results emphasize the current lack of mechanistic understanding for how *LIN28*/*let-7* mediates increases in HbF expression. Future studies of LIN28A, including examination of alternate model systems and determination of the minimal LIN28A levels required for its regulation of globin gene expression, are needed.

The sickling morphology of erythrocytes is due to polymerization of hemoglobin S with formation of intracellular fibers [31]. For decades, a combination of basic and clinical research has been aimed toward augmentation of HbF because of its sparing effects on sickle hemoglobin polymerization [5], [32]. We developed a novel approach for studies of the sickling phenomenon using culture-generated erythrocytes. During the terminal stages of differentiation, the erythroblasts were cultured in a 2% oxygen environment consistent with levels in bone marrow [17], [33]. Highly purified populations of enucleated cells were then imaged to demonstrate sickle morphologies. *LIN28A*-OE significantly reduced the morphological sickling in both donors' cells. While several factors are involved in the cellular sickling phenomenon [34], [35], increased HbF expression in *LIN28A*-OE cells is thought to play a critical role in reducing the morphological abnormalities.

In summary, *LIN28A*-OE reduced *let-7* miRNAs levels and increased HbF expression in erythroblasts cultured from CD34(+) cells from pediatric sickle cell anemia donors' blood at magnitudes similar to those previously reported using cells from healthy adults. The *ex vivo* culture model was explored in the context of tissue engineering to demonstrate reduction of the sickling phenomenon among the enucleated cells. As such, these data support further studies of the *LIN28*/*let-7* regulatory axis in erythroid biology with the eventual goal of identifying new therapeutic interventions for beta-hemoglobinopathies.

## Supporting Information

Figure S1 **Low oxygen culture conditions (2% oxygen) compared to 21% oxygen culture conditions increases erythroid enucleation of cultured CD34+ cells from healthy volunteers.** Enucleation was assessed by thiazole orange staining. Flow cytometry analyses of three independent healthy volunteers were performed at culture day 21. HV  =  healthy volunteer.
(TIF)

Figure S2 **Effect of** ***LIN28A*** **over-expression upon the expression levels of** ***BCL11A*** **.** ***BCL11A*** **mRNA levels were investigated by Q-RT-PCR quantitation of copy number per nanogram of complementary DNA (cDNA) (copies/ng cDNA) at culture day 14.** Open bars represent control and black bars represent *LIN28A* over-expression. C: empty vector control; OE: *LIN28A* over-expression.
(TIF)

Figure S3 ***LIN28A*** **over-expression increases the percentage of cells with high-levels of fetal hemoglobin.** Fetal hemoglobin staining was assessed by flow cytometry analysis of control and *LIN28A-OE* samples on culture day 21. Cell analyses from **(A)** Donor 1, **(B)** Donor 2, **(C)** Donor 3, **(D)** Donor 4 and **(E)** Donor 5 are shown. Fluorescence units (FU) for cells stained with the isotypic control antibody (IgG1) are shown in the shaded histograms. Fluorescence from the anti-HbF stained, control and *LIN28A-OE* transduced cells are demonstrated by dotted- and bold-lined histograms, respectively. The bar in the upper right of each panel denotes the fluorescence within the upper two decades (defined here and described in the text as high-level fluorescence).
(TIF)

Figure S4 ***LIN28A*** **over-expression in human sickle erythroblasts does not affect erythroblast differentiation and enucleation.** Flow cytometry analyses of **(A)** control and *LIN28A-OE* at culture day 14 and day 21 stained with anti-transferrin receptor (CD71) and anti-glycophorin A (GPA) antibodies. **(B)** Enucleation was assessed by thiazole orange staining of culture day 21 for control and *LIN28A-OE* cells. Data are representative of five independent research subjects. Control: empty vector control; *LIN28A-OE*: *LIN28A* over-expression.
(TIF)

Figure S5 **Microscopic fields of HbSS cells. Enucleated cells from Donor 4 (A) control and (B)** ***LIN28A-OE*** **, and Donor 5 (C) control and (D)** ***LIN28A-OE*** **.** The square in the first field from each image corresponds to the representative field shown in Figure 5. Control: empty vector control; *LIN28A-OE*: *LIN28A* over-expression.
(TIF)

Figure S6 **Enucleated erythroid cells from healthy HbAA donor.** Representative fields from **(A)** control (non-transduced cells) and **(B)** transduced cells. Culture conditions, cell imaging and counting procedures were the same as used for the sickle erythroid cells.
(TIF)
