# Supplementary figures and images for: LIN28A Expression Reduces Sickling of Cultured Human Erythrocytes
Source: PLoS One. 2014 Sep 4;9(9):e106924. doi: 10.1371/journal.pone.0106924 (PMC4154803; doi:10.1371/journal.pone.0106924)

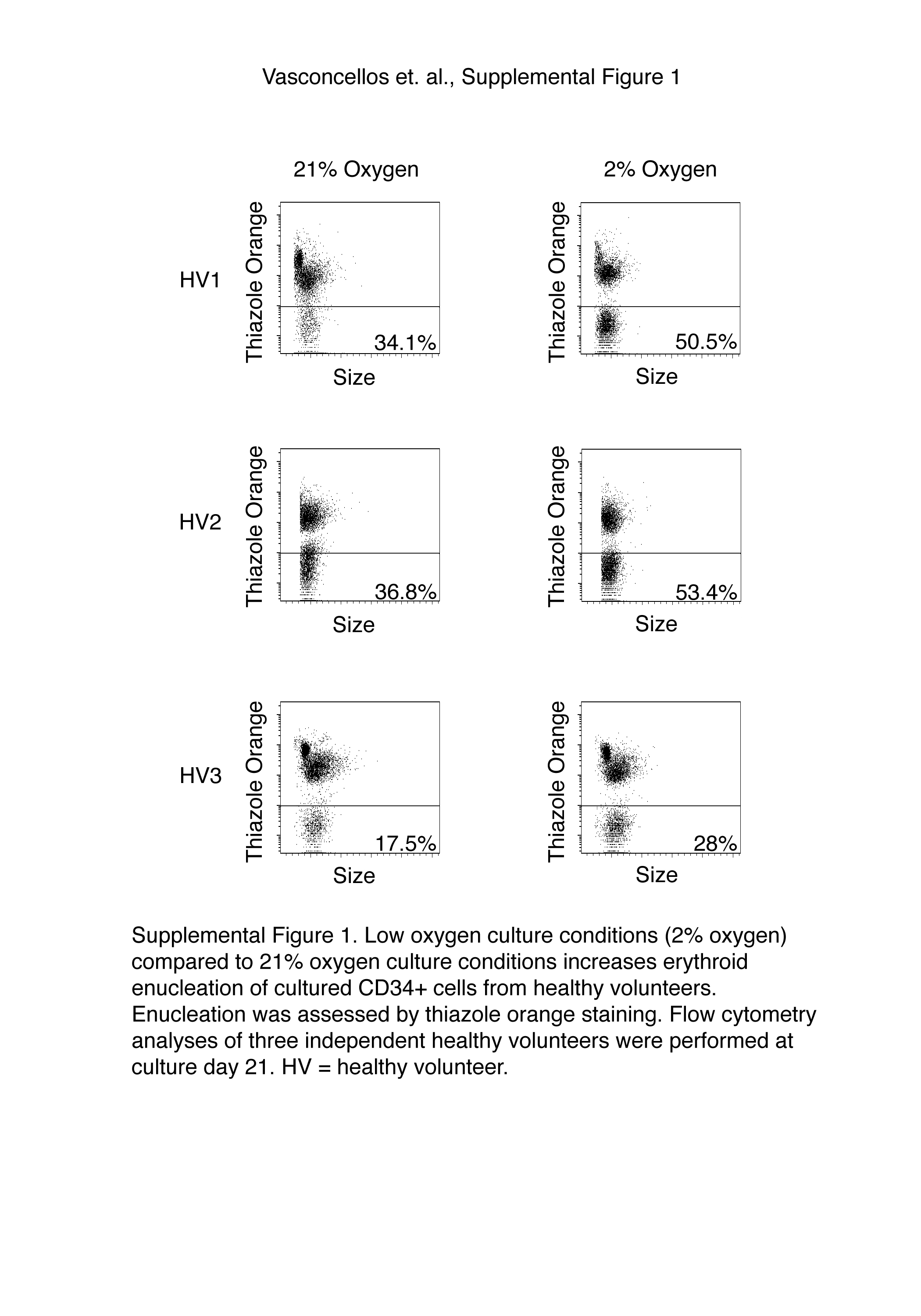

Supplement: Figure S1 — Low oxygen culture conditions (2% oxygen) compared to 21% oxygen culture conditions increases erythroid enucleation of cultured CD34+ cells from healthy volunteers. Enucleation was assessed by thiazole orange staining. Flow cytometry analyses of three independent healthy volunteers were performed at culture day 21. HV = healthy volunteer. (TIF) [file pone.0106924.s001.tif]

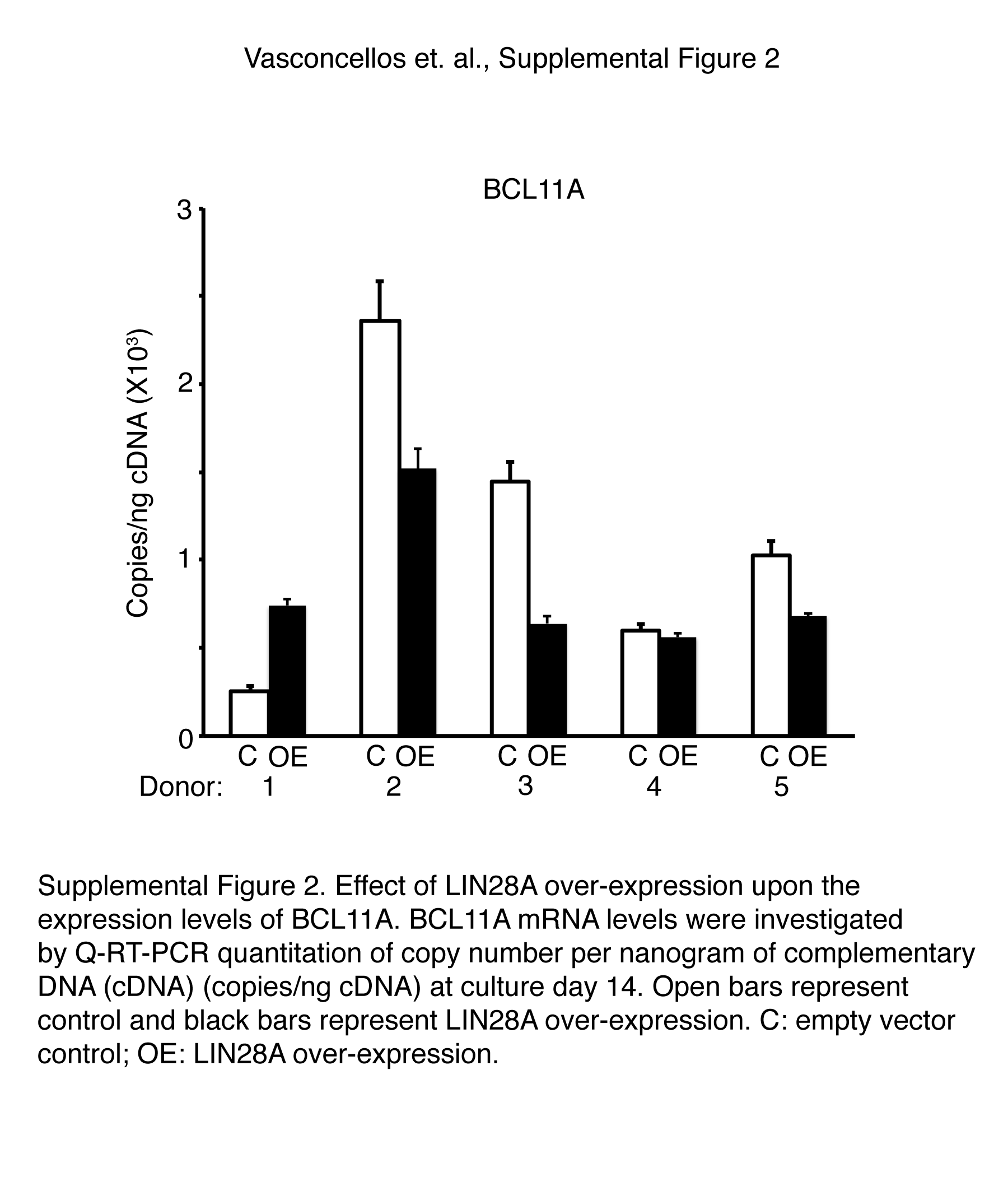

Supplement: Figure S2 — Effect of LIN28A over-expression upon the expression levels of BCL11A . BCL11A mRNA levels were investigated by Q-RT-PCR quantitation of copy number per nanogram of complementary DNA (cDNA) (copies/ng cDNA) at culture day 14. Open bars represent control and black bars represent LIN28A over-expression. C: empty vector control; OE: LIN28A over-expression. (TIF) [file pone.0106924.s002.tif]

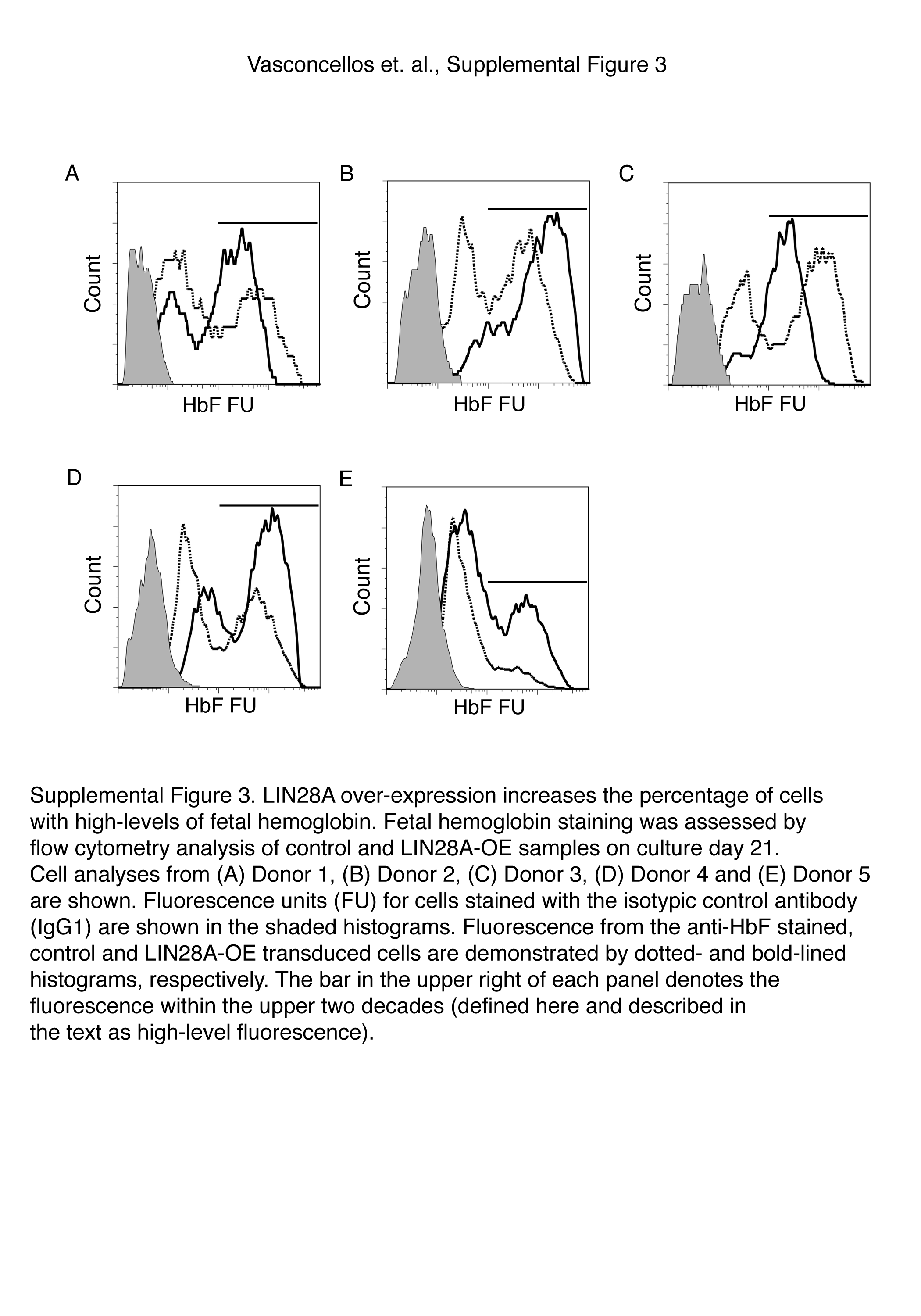

Supplement: Figure S3 — LIN28A over-expression increases the percentage of cells with high-levels of fetal hemoglobin. Fetal hemoglobin staining was assessed by flow cytometry analysis of control and LIN28A-OE samples on culture day 21. Cell analyses from (A) Donor 1, (B) Donor 2, (C) Donor 3, (D) Donor 4 and (E) Donor 5 are shown. Fluorescence units (FU) for cells stained with the isotypic control antibody (IgG1) are shown in the shaded histograms. Fluorescence from the anti-HbF stained, control and LIN28A-OE transduced cells are demonstrated by dotted- and bold-lined histograms, respectively. The bar in the upper right of each panel denotes the fluorescence within the upper two decades (defined here and described in the text as high-level fluorescence). (TIF) [file pone.0106924.s003.tif]

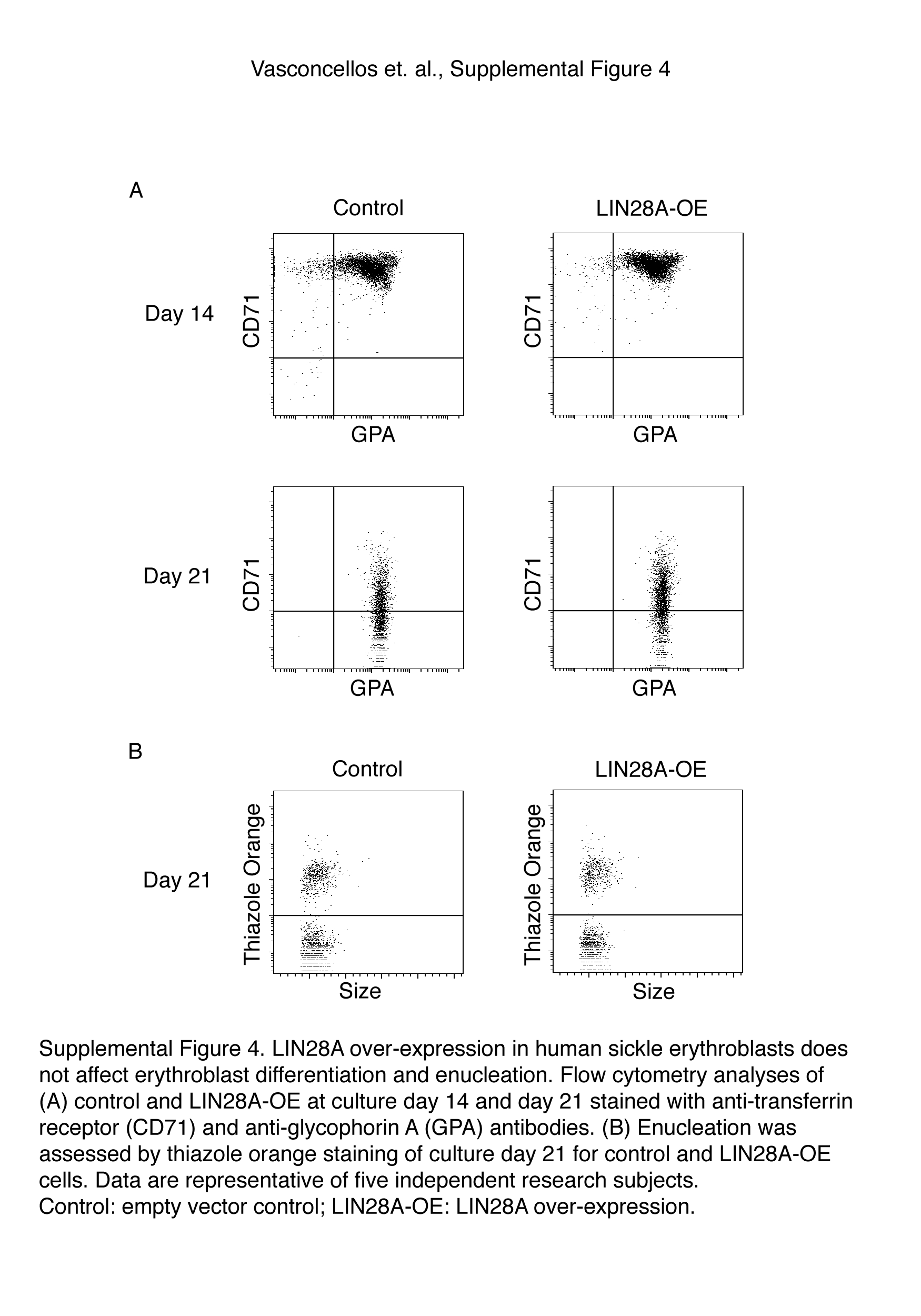

Supplement: Figure S4 — LIN28A over-expression in human sickle erythroblasts does not affect erythroblast differentiation and enucleation. Flow cytometry analyses of (A) control and LIN28A-OE at culture day 14 and day 21 stained with anti-transferrin receptor (CD71) and anti-glycophorin A (GPA) antibodies. (B) Enucleation was assessed by thiazole orange staining of culture day 21 for control and LIN28A-OE cells. Data are representative of five independent research subjects. Control: empty vector control; LIN28A-OE: LIN28A over-expression. (TIF) [file pone.0106924.s004.tif]

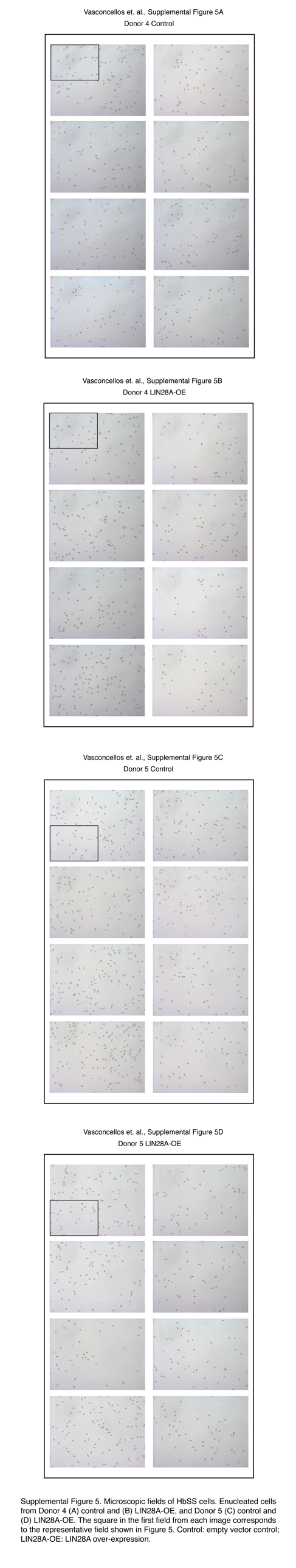

Supplement: Figure S5 — Microscopic fields of HbSS cells. Enucleated cells from Donor 4 (A) control and (B) LIN28A-OE , and Donor 5 (C) control and (D) LIN28A-OE . The square in the first field from each image corresponds to the representative field shown in Figure 5. Control: empty vector control; LIN28A-OE: LIN28A over-expression. (TIF) [file pone.0106924.s005.tif]

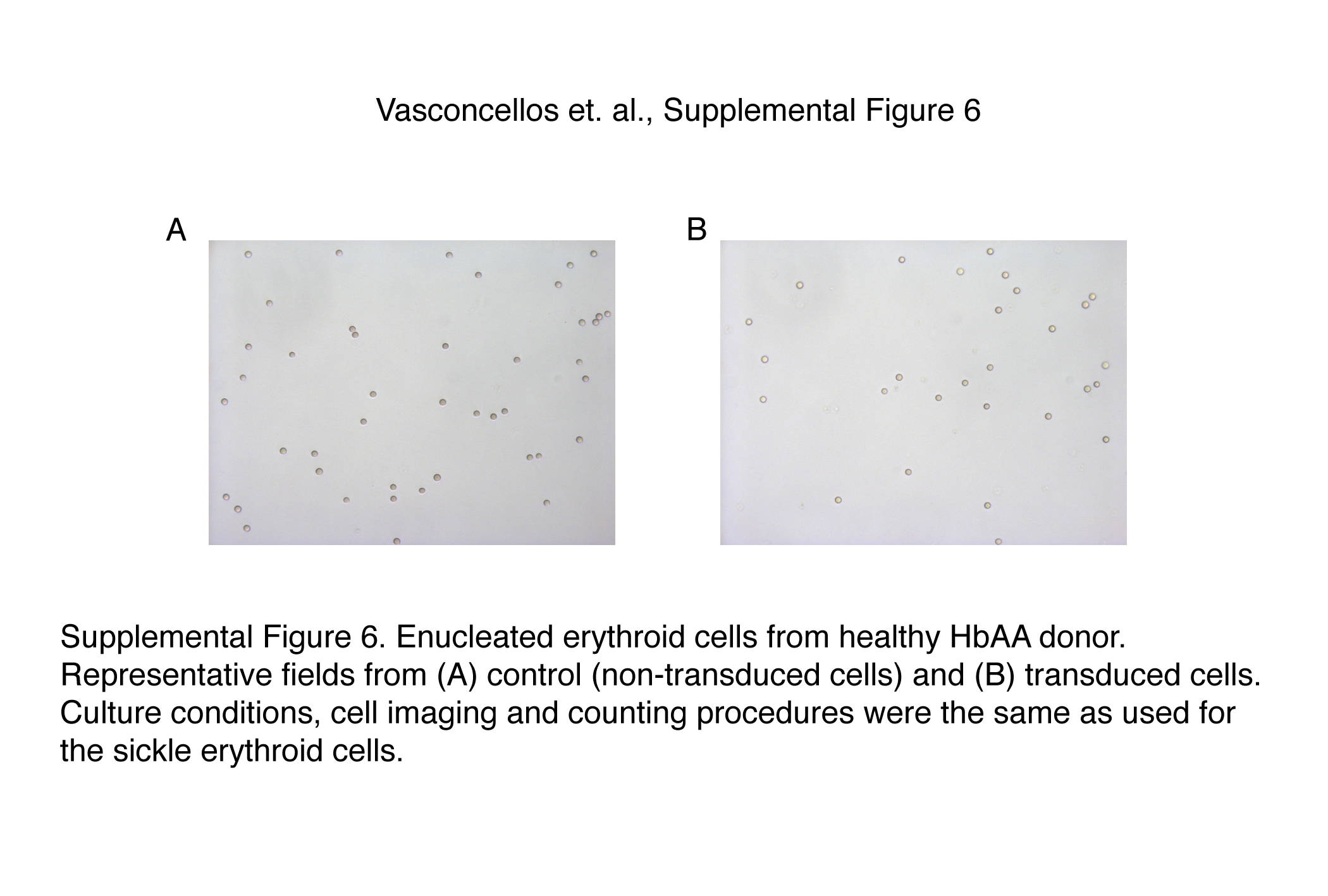

Supplement: Figure S6 — Enucleated erythroid cells from healthy HbAA donor. Representative fields from (A) control (non-transduced cells) and (B) transduced cells. Culture conditions, cell imaging and counting procedures were the same as used for the sickle erythroid cells. (TIF) [file pone.0106924.s006.tif]
